# Novel Concepts of Glioblastoma Therapy Concerning Its Heterogeneity

**DOI:** 10.3390/ijms221810005

**Published:** 2021-09-16

**Authors:** Gábor Hutóczki, József Virga, Zsuzsanna Birkó, Almos Klekner

**Affiliations:** 1 Department of Neurosurgery, University of Debrecen, H-4032 Debrecen, Hungary; aklekner@yahoo.com; 2 Department of Oncology, University of Debrecen, H-4032 Debrecen, Hungary; virga.jozsef@gmail.com; 3 Department of Human Genetics, University of Debrecen, H-4032 Debrecen, Hungary; birko@med.unideb.hu

**Keywords:** glioblastoma, liquid biopsy, invasion spectrum, oncotherapy

## Abstract

Although treatment outcomes of glioblastoma, the most malignant central nervous system (CNS) tumor, has improved in the past decades, it is still incurable, and survival has only slightly improved. Advances in molecular biology and genetics have completely transformed our understanding of glioblastoma. Multiple classifications and different diagnostic methods were made according to novel molecular markers. Discovering tumor heterogeneity only partially explains the ineffectiveness of current anti-proliferative therapies. Dynamic heterogeneity secures resistance to combined oncotherapy. As tumor growth proceeds, new therapy-resistant sub clones emerge. Liquid biopsy is a new and promising diagnostic tool that can step up with the dynamic genetic change. Getting a ’real-time’ picture of a specific tumor, anti-invasion and multi-target treatment can be designed. During invasion to the peri-tumoral brain tissue, glioma cells interact with the extracellular matrix components. The expressional levels of these matrix molecules give a characteristic pattern, the invasion spectrum, which possess vast diagnostical, predictive and prognostic information. It is a huge leap forward combating tumor heterogeneity and searching for novel therapies. Using the invasion spectrum of a tumor sample is a novel tool to distinguish between histological subtypes, specifying the tumor grades or different prognostic groups. Moreover, new therapeutic methods and their combinations are under trial. These are crucial steps towards personalized oncotherapy.

## 1. Introduction

**Figure 1:**
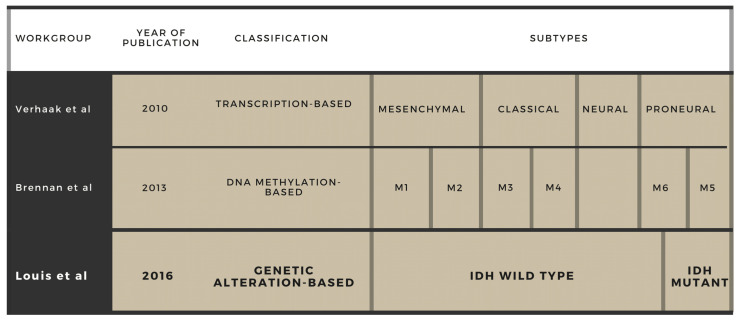
Molecular-based GBM classifications. Milestones in the classification of the glioblastoma subtypes (current WHO definition highlighted in bold).

Glioblastoma multiforme (GBM) is the most malignant tumor in the CNS and one of the tumors with the poorest prognosis considering other locations as well. It is incurable and inevitably fatal; hence, therapy can only hinder its progression and prolong survival with a tolerable quality of life. GBM represents 14.7% of all primary CNS tumors and 47.7% of all primary malignant CNS neoplasms. Its incidence rate increases with age, with the highest rate in individuals between the ages of 75 and 84 years, along with a considerable prevalence in the middle-aged population. Most of the diagnosed patients is usually aged 45–65. Younger age is reported to associate with a better outcome, and male patients have a better prognosis compared to female ones. Its aggressiveness results in a poor 5-year survival rate of 5.6%. Despite intensive research in the last decades, the OS still remains under expectations. During the course of the disease, the majority of patients experience some degree of impaired self-care, and a considerable number requires frequent medical care and special assistance, placing a significant social and economic burden onto the healthcare system. GBM can be characterized with vast intra- and inter-tumoral genetic heterogeneity. Indeed, its name, ’multiforme’, refers to the histological diversity seen in specimens. A total of four histologic criteria are needed for a diagnosis: microvascular proliferation, presence of necrosis, nuclear atypia and high mitotic activity. In the past, glioblastoma was the sole diagnosis for all that fulfilled these criteria; thus, this entity comprised excessively heterogeneous types. Treatment opportunities were highly limited and unitary as well: early results with irradiation were disappointing, which was slightly improved by chemotherapy. Intensive research in the field of molecular genetics has led to the discovery of certain chromosomes, genes and molecules involved in GBM tumor biology, which widened our knowledge of glioma diagnostics, estimating survival and measuring the effectivity of a certain therapy. Later on, it became clear that creating a molecular profile can describe a tumor entity more precisely since the molecular markers seem to be superior to the histological markers. Applying these novel markers in routine patient management transformed the classification of GBM from time to time, creating new entities while eradicating others [1]. The first milestone in the classification was the separation of primary (de novo) and secondary glioblastomas, having proved distinct molecular events that lie underneath the two groups. In the 1990s, with the help of emerging techniques such as PCR and allele analysis, scientists identified different molecular markers but at first these were not enough to clarify the multiple genetic changes. Later, microarray analyses revealed numerous genes of GBM that could be used as biomarkers in the diagnosis. In 2010, Verhaak et al. created transcription-based groups that were based on gene expression profiles, and now this classification is widely accepted [2]. The subtypes (proneural, neural, classical and mesenchymal) could be separated by distinct molecular defects that affect therapeutic response and survival. The proneural form occurs mainly in younger age and can be characterized with the IDH-1 mutation. After a revision in 2017, the neural type was found to arise from contamination of the original samples with non-tumour cells; hence, it was taken out from the classification [3]. The mesenchymal form, with its necrosis and neo-angiogenesis, has the worst prognosis. DNA methylation as an epigenetic change in GBM is a key process in tumor formation and progression via regulation of genomic functions. DNA methylation can provide biomarkers (e.g., MGMT, GATA6, CD81, DR4, CASP8 and CpG) for early diagnosis and prognosis. Discovering new epigenetic changes has allowed Brennan et al. to create six subgroups based upon the extent of the methylation (M1–M6) [4]. Frequent use of ‘omics’ technologies allowed whole-genomic analyses. The application of IDH-1 analysis with its tremendous predictive and prognostic value has led to the current WHO classification presented by Louis et al.: IDH-wild type/IDH mutant/NOS [5] (Figure 1). IDH-mutant GBM patients have a significantly higher overall survival and are more responsive to the chemotherapeutic agent temozolomide compared to patients with wild-type GBM.

## 2. The Role of Tumor Heterogeneity in Treatment Ineffectiveness

Heterogeneity is a widely known feature of GBM, as implied by its name: ’multiforme’. Numerous subclones develop in the early stage due to rapid mitoses [6]. As the process continues, determining the actual genomic status is barely possible. This dynamic heterogeneity is at least partially responsible for the resistance to combined neuro-oncological therapy. Therefore, it is difficult to set up molecular-level diagnosis and targeted oncotherapy, which would be the basis of an effective treatment. Diagnosis greatly depends on the location of the sampling and the numerous subclones can lead to misgrading or wrong genomic status determination. To reduce this uncertainty, there are two main trends: (1) combined therapy against multiple targets and various parallel mechanisms aiming at different subclones can increase treatment effectivity; and (2) instead of using a local sample, the molecular signature of the entire tumor can be identified through liquid biopsy [7]. A great advantage of liquid biopsy is that it can be repeated multiple times with minimal risk; thus, the rapid genetic change of the tumor can be discovered early, even before the progression or recurrence is visible on MRI [8]. Frequent sampling leads to prompt discovery of the changing heterogeneity and oncologic treatment can be adjusted properly. The combination of these two approaches results in proper molecular pathological diagnosis and personalized complex oncotherapy. In this review, we focus on the novel treatment strategies that combat tumor heterogeneity.

## 3. Current Therapeutic Methods

**Figure 2:**
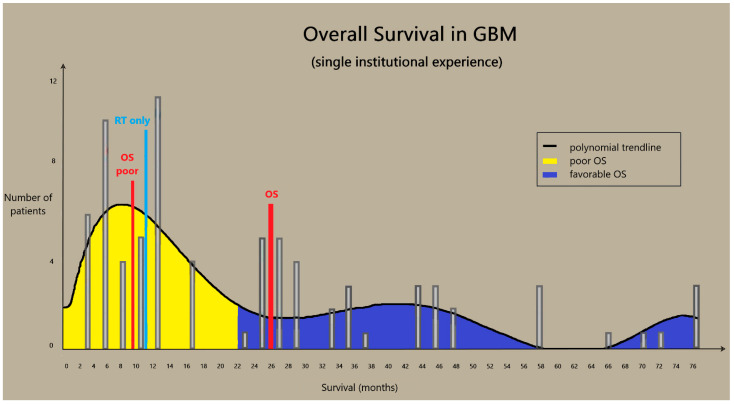
Overall survival in GBM (single institutional experience). Overall survival of patients treated (at least with concurrent chemo irradiation) with glioblastoma at our institution. The polynomial trend line shows three peaks. The first represents patients with poor prognosis—their median overall survival (OS poor) do not differ significantly from those who were treated only with radiotherapy (RT only) prior to Stupp era. The second and third peaks represent patients with a favorable prognosis. OS: Median overall survival of all examined patients.

The overall survival (OS) of GBM patients without postoperative treatment is 3–6 months. The introduction of radiotherapy (RT) increased the OS rates to 9–12 months. Temozolomide (TMZ) has been added to RT, resulting in yet another increase in survival. Despite this progress, the treatment protocol for GBM has remained essentially unchanged in the past years despite the enormous progress in diagnostics. As of 2006, patients receive standard post-surgical concurrent chemo-radiotherapy and subsequent temozolomide monotherapy regardless of the diagnostic subgroups [9]. The spread of the Stupp protocol was a great leap in OS and progression-free survival (PFS); this improvement has been well-proven worldwide, as well as in our database. Then, the introduction of bevacizumab (BEV) further improved survival rates [10]. According to our previous findings, OS was statistically independent of age, gender, size, side, and location of the tumor, as well as to the extent of surgery among patients who received the standard treatment by Stupp. Patients with an insufficient Karnofsky Prognostic Score (KPS < 70) received only palliative irradiation and their life expectancy was unfortunately very low. Concerning those with good general performance status, when concurrent therapy could be started, a vast difference in survival was found and attributed to distinct radio-chemo sensitivity, resulting in two groups: responder (PFS = 13.4 ± 7.5 months, OS = 25.7 ± 7.4 months) and non-responder (PFS = 4.5 ± 2.3 months, OS = 10.2 ± 4.2 months). Non-responder means that the tumor is not radio-chemo sensitive, and progression or recurrence occurs much earlier, despite concurrent treatment (Figure 2). This group comprises approximately 50% of the patients [11]. The lack of effectiveness of the therapy can be attributed to tumor heterogeneity and the GBM’s invasive behavior. Its invasive nature makes radical resection and radiosurgery impossible, while heterogeneity reduces the success of chemotherapy. Multiple mutations at any level of the three major pathways (RTK/RAS/PI3K, P53 and RB) result in intra-tumor and inter-tumor heterogeneity through the rise of chemo-resistant subclones. Besides heterogeneity, the other major intrinsic feature of GBM is that glioma cells can migrate across large distances, and thus the tumor becomes effectively borderless. Unlike cerebral metastases that are usually well demarcated and can be separated from the non-tumor brain, malignant gliomas bear high invasive potential. Glioma cells interact with each other and the components of the extracellular matrix, and can migrate far beyond the visible tumor border into the peritumoral tissue [12]. Radio- and chemotherapy hinder cell proliferation but do not have a significant anti-invasive effect. Radical tumor elimination is not feasible due to heterogeneity and cell migration. Cyto-reductive treatments have only a temporary effect and recurrence is inevitable. In recurrent tumors, therapy-resistant subclones emerge, new genetic alterations are present, and therefore oncotherapy becomes ineffective, resulting in unmanageable progression that leads to rapid patient death [13]. Examinations that can modify the treatment plan already exist in routine molecular pathological practice (e.g., test for MGMT methylation, 1p19q co-deletion), but molecular profile-based targeted therapy is still lacking.

## 4. New Diagnostic Methods for Personalized Therapy

Since current histological analysis is not suitable for targeted therapy, a new concept using novel diagnostic tools is needed. Tissue samples represent only a fraction of the whole tumor at a certain of time. Diagnostic inaccuracy can be attributed to heterogeneity and dynamic changes. 

### 4.1. Liquid Biopsy

A key step in the management of glioma is the difficulty in obtaining tissue samples eligible for molecular analysis. These samples can only be obtained by surgical resection or stereotactic biopsy with a considerable risk of life-threatening complications. Besides the high risk these procedures pose, the size of the tumor samples is often limited and is not always representative of the whole tumor. Only a fraction of the whole molecular profile of the tumor can be determined, especially in cases of biopsies due to intratumoral heterogeneity. Chances of monitoring disease progression or therapeutic responsiveness are also restricted, since repeated invasive biopsies are not reasonable. Blood, cerebrospinal fluid and urine samples have been found to contain tumor components in different forms: cells, extracellular vesicles and circulating cell-free nucleic acids. A growing number of research studies supports the examination of these exosomes, DNA and RNA particles. These so called “liquid biopsy” samples can represent the entire tumor tissue and possess enormous diagnostic, prognostic and predictive potential. Their functions have been proved to contribute to the sophisticated background communication between various cell types, stroma, blood vessels, secreted factors and surrounding ECM. Liquid biopsy is a rapid and inexpensive way of obtaining the relevant information about tumors and can be performed several times during the clinical course of the disease [14]. Furthermore, integrating analyses of EVs and related cf-NAs in clinical practice might also help to establish a diagnosis in a non-invasive manner, and complex oncotherapy could be indicated in the future without high-risk neurosurgical interventions [15]. Despite the significance of this novel method, it also has its own limitations. There is still no consensus among researchers about the type of nucleic acids, biological fluids or pre-analytical/analytical techniques that gives the best results. On the other hand, liquid biopsy can help gain accurate information about the newly developed genetic defects of GBM or the effectiveness of anti-tumor therapy. For these purposes, new highly sensitive and rapid diagnostic tools have been developed and are proposed for a more accurate diagnosis: next-generation sequencing (NGS) has revolutionized genomic-level analyses [16]. Digital droplet PCR (ddPCR), with its truly quantitative possibilities, supports liquid biopsy perfectly [17]. 

Glioma stem cells (GSCs), as a cellular origin of GBM, can be found in the necrotic and hypoxic center of the tumor and are believed to be the main source of heterogeneity and therapy resistance. They exist in two forms based on the cellular origin: proneural and mesenchymal GSCs. Proneural cells are less aggressive and can be found in secondary GBMs, while primary, highly invasive and fast-growing glioblastomas derive from the mesenchymal types [18]. Culturing GSCs can create specific in vitro ‘glioma organoid’, which can be the basis for whole-genome analysis and personal treatment [19].

#### 4.1.1. Circulating Nucleic Acids

Nucleic acids (both DNA and RNA) derived from tumor cells circulate in a cell-free form, are attached to lipid/protein structures or are found in circulating extracellular vesicles (EVs). These EVs can elicit different types of interactions with nearby cells (molecular release, direct EV-target cell surface contact or fusion). Circulating DNA types include cell-free DNA (cfDNA), circulating tumor DNA (ctDNA) and mitochondrial DNA (mtDNA). CfDNA has a 150–200 base-pair-long, double-stranded structure and a 10–15 ng/L concentration in healthy human plasma. In cancer patients, the plasma level is significantly increased, and the composition was also shown to be changing over time. CtDNA derived from tumor cells is the major cfDNA fraction [20]. Mutations that can be detected in the ctDNA carry remarkably valuable information about the genetic change in the tumor tissue. The plasma level of ctDNA is a perfect indicator of therapeutic response. An extremely low level and short half-life of ctDNA makes it difficult to be used in diagnostics, especially in GBM, but with the help of highly sensitive and tumor-specific NGS panels, BRAF/IDH1/IDH2 mutations and ERBB2/MET/EGFR/PDGFRA amplifications have already been determined [21]. In case of primary brain malignancies, high levels of ctDNA can be measured in the CSF. Quantitative and qualitative alterations in mtDNA could be the first sign of tumors as well. Changes in mtDNA starts early in the premalignant stage, and a high level indicates poor prognosis. MtDNA may take a prominent role in the early diagnosis of new and recurrent GBM in the future [22]. 

Circulating RNA types include messenger RNA (mRNA), short and long non-coding RNA (snc and lncRNA), micro-RNA (miRNA) and circular RNA (circRNA). MiRNAs have the most prominent role; these 19–25 bp long nucleic acids are excellent biomarkers and are abundant in most bodily fluids. They are great examples of epigenetic mechanisms regulating the expression of multiple target genes by forming a “silencing complex” [23]. MiRNAs act as post-transcriptional inhibitors either by degrading specific mRNAs or by inhibiting translation. Intervention in signaling pathways affect cell metabolism and tumorigenesis. RTK/RAS/PI3K is the principal pathway in GBM, and the expression level of EGFR is influenced by many miRNAs in direct (e.g., miR-7, miR-219-5p) and indirect (miR-21) ways [24,25]. Targeting kRAS, the level of miR-134 is highly decreased while the level of miR-9 is increased in GBM. Other miRNAs that affect this signaling pathway are miR-542-3p, miR-503, miR-26a, miR-10a/10b, miR-494-3p and miR-1908. It has also been demonstrated that miRNAs are stable, due to their considerable resistance to RNAse activity. In glioblastoma the miR21, miR182, miR210, miR221, miR222 and miR454 levels are increased, while the miR128 and miR342 levels are decreased, and their amount show strong correlation with the tumor volume. LncRNAs take part in tumor progression via modifications in the signaling pathways: low HOTAIR and high GA5 levels are good prognostic factors in GBM. SncRNAs (circRNA and miRNA) are prone to form miRNA ’sponges’ that facilitate glioma genesis and progression [26].

#### 4.1.2. Exosomes and Tumor Cells

Cells can release vesicular structures with a luminal center covered by the phospholipid bilayer of the plasma membrane. They are heterogeneous and three major subgroups could be distinguished: exosomes, microvesicles and apoptotic bodies. Exosomes are extracellular vesicles with a dimension of 40–150 nm in diameter. Nucleic acids, proteins, lipids and metabolites are enclosed in a double lipid membrane layer escaping degradation. Exosomes derived from tumor cells help cell communication by establishing its micro-environment. This communication can facilitate the development of resistance to irradiation or chemotherapy (e.g., TMZ). Thus, elimination of tumor exosomes from serum has immense therapeutic potential [27]. The role and potential clinical use of circulating tumor cells (CTCs) is yet unclear because of their extremely low levels in blood (1 CTC out of 109 blood cells) and their difficult extraction [28].

## 5. Invasion Spectrum 

Tumor cells can migrate to neighboring tissue and invade the peri-tumoral brain. Extensive invasion of cancer cells to the adjacent parenchyma makes surgical removal impossible and leads to inevitable local recurrence. Previous studies have demonstrated that the extracellular matrix (ECM) is altered in composition in various cancer types. Invasion activity of glioma cells is based on interaction with well-defined ECM components. It is a complex process with transmembrane receptors, extracellular ligands and synthetizing and degrading enzymes [29]. These differences and changes play a major role in glioma invasion and result in different invasiveness. The expression pattern of ECM molecules is a distinctive feature of tumor grades. Non-invasive tumors show only slight differences in invasion-related ECM components. The key molecules (e.g., brevican, cadherin-12, fibronectin and integrin-β1) correlating the most with tumor grade have been identified. Glioma samples can be separated based on their expressional pattern using statistical classifiers. Therefore, the ECM composition seems to be typical of various cancer types and the expression levels of its components have been linked to tumor invasion. Identifying an unknown sample’s grade using these statistical classifiers is possible as well. Differences in the ECM of non-tumor brain and glioblastoma have already been proven. Normal brain and glioma expression patterns, along with low-grade astrocytoma and glioblastoma samples differ the most [30]. Determining the invasion-related molecules’ expression profile provides additional information regarding the tumor’s clinical behavior and prognosis. The invasion spectrum can also be used as a considerably accurate prognostic factor in glioblastoma [31]. The characteristic molecular profile correlates with the survival of patients, and it is possible to accurately identify the favorable and poor prognostic groups using tumor samples, the method having a higher accuracy in patients with a poor prognosis [32]. Identifying molecules playing a key role in glioma invasion could uncover potential therapeutic targets and gain predictive information in the future.

## 6. New Therapeutic Directions

**Figure 3:**
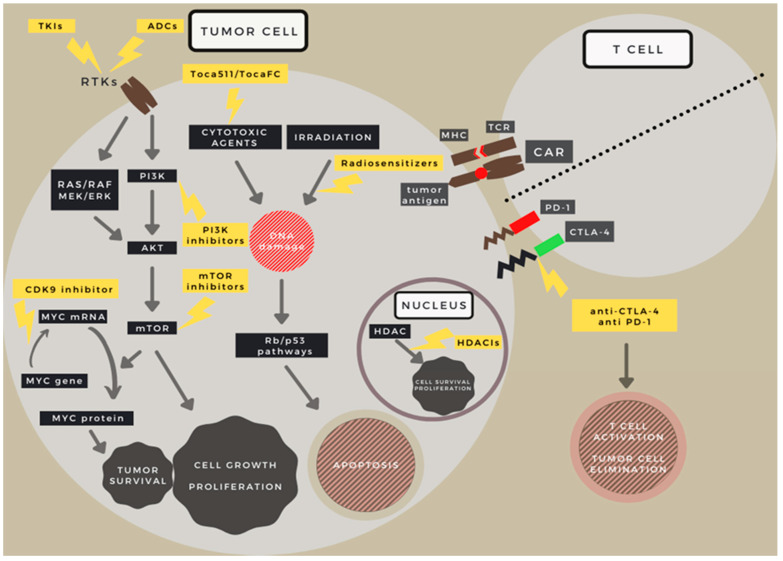
Targets of various drugs used in GBM treatment trials on a schematic depiction. Target locations of new therapeutic directions in GBM. RTK (-RAS-PI3K-mTOR), TP53 and RB pathways and the tumor cell–T cell connections are visualized schematically. Abbreviations: TKIs: Tyrosine kinase inhibitors; ADCs: Antibody drug conjugates; CDK9: Cyclin Dependent Kinase 9; RTKs: Receptor tyrosine kinases; RAS: Rat sarcoma virus protein family; Raf: Serine/threonine-specific protein kinase family; MEK: Mitogen-activated protein kinase; ERK: Extracellular signal-regulated kinase; PI3K: Phosphoinositide 3-kinase; AKT: Serine/threonine protein kinase family; mTOR: mechanistic target of rapamycin; Myc: Regulator gene and proto-oncogene family; HDAC: Histone deacetylase; HDACIs: Histone deacetylase inhibitors; TCR: T cell receptor; MHC: Major histocompatibility complex; CAR: Chimeric Antigen Receptor; PD-1: Programmed cell death protein 1; CTLA-4: Cytotoxic T-lymphocyte-associated protein 4; Rb: Retinoblastoma; p53: Cellular tumor antigene p53.

**Figure 4:**
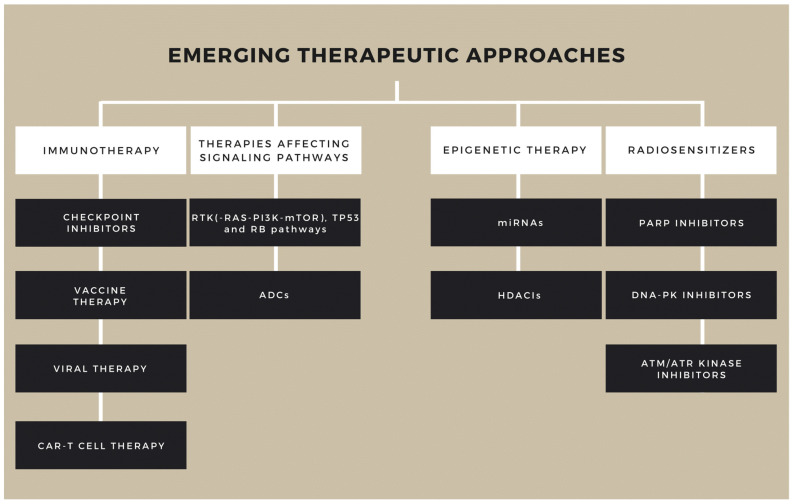
Novel treatment modalities in glioblastoma. At present the gold standard therapy is used worldwide, but promising new agents are under trial. Abbreviations: ADCs: Antibody drug conjugates; RTKs: Receptor tyrosine kinases; RAS: Rat sarcoma virus protein family; PI3K: Phosphoinositide 3-kinase; mTOR: mechanistic target of rapamycin; HDACIs: Histone deacetylase inhibitors; CAR: Chimeric Antigen Receptor; Rb: Retinoblastoma; p53: Cellular tumor antigene p53; miRNAs: microRNAs; PARP: Poly (ADP-ribose) polymerase; DNA-PK: DNA-dependent protein kinase; ATM/ATR: Ataxia telangiectasia mutated/ATM and RAD3-related.

Searching for new therapeutic targets aims to inhibit multiple signaling pathways at the same time and to hinder the proliferation of therapy-resistant subclones. There are numerous studies targeting the elimination GBM via different mechanisms, but no breakthrough in treatment has been reached yet (Figure 3) [33]. The main obstacles are lack of selectivity, variable (usually low) effectivity, high toxicity, activation of alternative pathways in targeted therapies, inability to reach sufficient concentration levels in the tumor tissue (due to blood brain barrier), and strong tumoral immunosuppressive effect [34]. Using multiple agents that proved to be effective in other malignancies can be favorable in the battle against GBM (Figure 4).

### 6.1. Supplemented Standard Treatment

Besides irradiation, only two antitumor agents (TMZ, BEV) and pulsating electric tumor-treating fields (TTF) have FDA approval for GBM at present [35]. TTF are low-intensity, alternating electric fields that can arrest the cell cycle. Some research groups add other agents to the current standard therapy (e.g., irinotecan or lomustin in addition to bevacizumab). In the BELOB study, the administration of lomustin besides bevacizumab in recurrent GBM significantly increased OS [36]. Neoadjuvant pembrolizumab treatment in recurrent, operable glioblastomas also yielded encouraging results [37]. 

### 6.2. Immunotherapy

Glioblastoma is an immunologically ’quiet’ disease, which means that an immunosuppressed tumor microenvironment is provided with minimal antigene presentation, different antigene escape mechanisms and direct immunosuppression [38]. Immunotherapy uses the tumor-eliminating potential of the personal immune system via different ways. Creating an intense inflammatory intracranial response can be highly effective but also poses a danger, which implies severe side-effects, even life-threatening conditions. 

#### 6.2.1. Checkpoint Inhibitors (ICIs)

Tumor cells are able to avoid cellular immune response and elimination by the following mechanism: certain antigens (e.g., CTLA-4, PD-1) that are expressed on the surface of T-cells bind to their corresponding ligands located on the tumor surface. This leads to reduced cytotoxic T-cell activation. Blocking these antigens selectively by MABs stimulates an immune response. Clinical trials with anti-CTLA4 (nivolumab, ipilimumab) or anti-PD1 (pembrolizumab) show encouraging preliminary results [39,40]. The methylated tryptophan indoximod also has checkpoint inhibitory activity by inhibiting the enzyme indoleamine 2,3-dioxygenase (IDO), which plays a crucial role in T cell arrest and anergy.

#### 6.2.2. Vaccine-Based Therapy 

Training the immune system with vaccination is a very hot topic nowadays, and it can be a useful tool against tumors as well. Tumor-associated and tumor-specific antigenes (TAA and TSA) generate the immune response: a protein vaccine against EGFRvIII (rindopepimut) has promising early results [41]. Another potential target is survivin from the apoptosis inhibitor protein family (SurVaxM) [42]. Besides protein-based vaccines, preliminary results with activated autologous dendritic cells (e.g., DCVax) are also hopeful. HSPPC-96 (heat shock protein peptide comlex-96) is the pioneer representative of personalized cancer vaccines. It is made by training the patient’s immune system to recognize the gp96 heat shock protein and its related proteins that are extracted from his or her own tumor tissue [43].

#### 6.2.3. Viral Therapy

Using viral vectors with the ability to integrate into the host tumor genome is another type of immunotherapy. These vectors contain selected genes that code enzymes or other proteins with a potential lethal effect on tumor cells. Toca 511 and Toca FC is a combination of gene therapy and a prodrug. Retroviral vector Toca 511 (vocimagene amiretrorepvec) contains gene-encoding cytosine deaminase. Toca FC is a prodrug of 5-fluorouracil, a well-known chemotherapeutic agent that can cross the blood–brain barrier and transforms locally to 5-fluorouracil [44]. 

Oncolytic viruses are genetically engineered viral particles that can infect and dismiss tumor cells selectively while leaving adjacent brain tissue intact. Neural stem cells (NSCs) have the potential to carry oncolytic viruses to the target, thus increasing effectivity. Three virus families have gained significance so far: adenoviruses (e.g., Delta-24-RGD), herpesviruses (e.g., Talimogene laherparepvec) and recombinant polio-rhinoviruses (e.g., PVSRIPO). Delta-24-RGD can elicit antitumor effects alone, but it also has promising results in combination with pembrolizumab [45]. Trends today hint that viral therapy is more of a complementary therapy, rather than a sole treatment option. 

#### 6.2.4. CAR-T Cell Therapy

Genetically modified autologous T cells are armed with specific receptors that can attach to tumor antigens and implement a lethal cytotoxic attack. These cells with chimeric antigen receptors are called CAR-T cells. Possible tumor antigens can be EGFRvIII, HER2 and IL13RA2 [46]. 

### 6.3. Signaling Pathway-Focused Therapies

There is vast potential in immunotherapy, but promising new attempts in other treatment modalities are also notable. Under physiological conditions, signal transduction cascades take part in everyday cell life, affecting proliferation, differentiation, mobility, cell–cell communication and survival. Tumors use these processes for excessive proliferation, abnormal angiogenesis or to evade apoptosis. In GBM, usually three major signaling pathways are dysregulated. Due to tumor heterogeneity, all the pathways can be affected in the same patient [47]. Members of the RTK (-RAS–PI3K–mTOR) complex cascade are proto-oncogenes promoting cell migration, proliferation and survival. Therefore, dysregulation leads to uncontrolled proliferation, long-distance migration, and abnormal survival. Hallmarks of the other two pathways, the TP53 and RB pathways, are tumor-suppressor genes. Their protective role enables cell cycle arrest and apoptosis; thus, impeding them means excessive proliferation and immortality. Intervention in different levels of the pathways has reached success in multiple types of cancer; however, none of them have been approved for patients with GBM. One possible reason may be the presence of the blood–brain barrier, but activation of parallel pathways also contributes to the lack of success. RTK (-RAS–PI3K–mTOR) is the most emphasized signaling pathway since the majority of GBMs bear at least one RTK family (EGFR, VEGFR, PDGFR, etc.) mutation. Small molecule TKIs (e.g., erlotinib) or MABs (e.g., nimotuzumab) can selectively block single or multiple (e.g., regorafenib) TK receptors [48,49,50]. Other agents that are under investigation in glioblastoma effecting this pathway are PI3K inhibitors (e.g., buparlisib, GDC-0084), dual PI3K/mTOR inhibitors (e.g., dactolisib) or mTOR inhibitors (e.g., AZD8055) [51,52]. Myc is a transcription factor that takes part in many cellular functions and controlled by multiple pathways. In glioblastoma, highly elevated myc levels can be detected [53]. The selective CDK9 inhibitor zotiraciclib can effectively decrease the myc levels and seems to be effective in combination with temozolomide [54].

#### ADCs

Cell surface antigenes (like TK receptors) expressed on the surface of glioma cells are potential targets of antibody-drug conjugate (ADC) therapeutics. ADCs are molecules with intrinsic antitumor effects bound to a specific antibody (against a surface antigen) and delivered directly to the tumor cells. EGFR appears to be a perfect antigen and the antibodies could deliver cytokines, antimitotic agents, bacterial toxins or radioactive isotopes [55,56,57]. Immunocytokines are ADCs that are immunotherapeutic agents as well. They use the pro-inflammatory potential of the selected cytokine. Clinical trials with L19TNF are promising with surprisingly low toxicity levels, but it seems that a better response can be reached by combining L19TNF with other chemotherapeutic agents, e.g., ICIs [58].

### 6.4. Epigenetic Therapy 

Searching for novel ways of intervention at the genomic level has led to the development of epigenetic therapy. Epigenetic mechanisms are deregulated in GBM. Modifying gene expression, transcription and translation has the potential of the inhibition of oncogenes and supporting suppressor genes by impeding proliferation, provoking cell cycle arrest and promoting apoptosis. Besides miRNAs that also can have epigenetic effects, histone deacetylase inhibitors (HDACIs) are potential candidates to hinder glioma cell proliferation. There is ongoing research with vorinostat and panobinostat in glioblastoma, mostly in combination with another type of drug [59].

### 6.5. Radiosensitizers

Radioresistance to ionizing radiation is a well-known feature of glioblastoma. Different underlying mechanisms have been identified, including microRNAs, tumor heterogeneity, glioma stem cells, tumor microenvironment, hypoxia, metabolic alterations and DNA damage/repair. Radiosensitizers are used to increase the efficacy of irradiation without increasing the dose of radiotherapy, which elevates the likelihood of side effects and damage to the normal brain tissue. Among the different mechanisms, the most important seems to be DNA damage response (DDR). PARP enzymes repair DNA damage, reinforce genome stability and hinder apoptosis. Olaparib is the first PARP inhibitor that has already reached encouraging results in breast and ovarian cancers and is now under investigation in GBM with veliparib and fluzoparib [60,61]. DNA-dependent protein kinase (DNA-PK) plays a crucial role in detection and repair of DNA double-strand breaks. The DNA-PK inhibitors AZD7648 and NU7441 are promising efficient sensitizers of radiation-induced DNA damage [62]. Other key regulators of DNA damage repair and genome stability maintenance are ATM/ATR kinases. Molecular inhibitor of ATM AZD1390 is currently in a phase I trial in GBM patients [63,64]. 

### 6.6. Novel Radiotherapy Approaches

Ionizing radiation is a well-known ‘ab ovo’ treatment modality of glioblastoma, which was used firstly as a sole option. Radioresistance and side effects that ruin QoL resulted in worse outcomes after radiation treatment in the past. Better instrumentation, the application of radiosensitizers and improvements in imaging technologies have led to the rebirth of radiation therapy. A constant upgrade in conformality and novel modalities deliver patient- and tumor-optimized radiation that spare normal tissues and minimize side effects. The evolution of increasing accuracy started with whole-brain radiotherapy (WBRT) in the beginning, which was later transformed to focal brain radiotherapy (FBRT). The development of three-dimensional techniques, such as intensity-modulated radiation therapy (IMRT) and volumetric arc radiation therapy (VMAT), allowed the delivery of increased doses sparing non-tumor tissue [65]. Radiosurgery (gamma knife, Zap-X) further increased conformality and reduced the received dose of tumor-adjacent tissues and OARs [66]. Maximizing the dose drop at the tumor margin has led to the trial of particle therapies. Currently, there is intensive research with proton beam therapy (PBT) and carbon ion radiotherapy (CIRT) [67,68]. 

## 7. Summary

The currently accepted standard therapy of GBM is maximal safe surgical resection followed by TMZ-based concurrent radio-chemotherapy. In case of recurrence, repeated surgery (if possible) and/or administration of bevacizumab are options. Our current molecular genetic knowledge highly surpasses the treatment options. In the past, glioblastoma was believed to be a single entity, but using our understanding of its genetic composition, GBM can now be divided into several distinct subtypes. These subtypes have excessively different diagnostic, predictive and prognostic potentials and can also be discriminated by their treatment targets and response. Liquid biopsy, as a new and emerging diagnostic tool, can perform molecular mapping of the heterogeneous tumor tissue and can follow the dynamic molecular changes during oncotherapy. It is clear that novel combined and personalized multi-target therapies are needed to further improve treatment outcomes. Most of the agents have already proved to be effective in other tumor types. In the future, the goal will be to match the combination of the available therapies with the subtype of the tumor. In general, there are a lot of ongoing studies, with promising early results, but the present therapy remains the gold standard. The most crucial criterion for prolonged survival is a good performance status of the patient after tumor resection so that nonsurgical oncotherapy can be started. The peritumoral invasion of glioblastoma makes complete tumor resection practically impossible; therefore, the previous surgical strategy of “maximal extension of tumor resection” is suggested to be replaced by “maximal safe tumor resection”. The effectiveness of antitumor therapy mainly depends on the radio- and chemo-sensitivity of the tumor cells, which shows great variation. To overcome this, a new treatment strategy could be the concept of combined therapy, with addition of anti-invasion agents to the currently used cyto-reductive, anti-proliferative agents. As a result, the demarcated tumor could be removed more radically by surgery or treated with stereotactic radiosurgery/radiotherapy. Since these treatment modalities can be repeated multiple times, recurrent tumors could be also treated effectively, which may prolong survival times.
